# A Pap1–Oxs1 signaling pathway for disulfide stress in *Schizosaccharomyces pombe*

**DOI:** 10.1093/nar/gkw818

**Published:** 2016-09-22

**Authors:** Yumei He, Yan Chen, Wen Song, Lei Zhu, Zhicheng Dong, David W. Ow

**Affiliations:** 1 Plant Gene Engineering Center, South China Botanical Garden, Chinese Academy of Sciences, Guangzhou 510650, China; 2 University of Chinese Academy of Sciences, Beijing 100049, China; 3 Plant Gene Expression Center, USDA/UC Berkeley, Albany, CA 94710, USA

## Abstract

We describe a Pap1–Oxs1 pathway for diamide-induced disulfide stress in *Schizosaccharomyces pombe*, where the nucleocytoplasmic HMG protein Oxs1 acts cooperatively with Pap1 to regulate transcription. Oxs1 and Pap1 form a complex when cells are exposed to diamide or Cd that causes disulfide stress. When examined for promoters up-regulated by diamide, effective Pap1 binding to these targets requires Oxs1, and vice versa. With some genes, each protein alone enhances transcription, but the presence of both exerts an additive positive effect. In other genes, although transcription is induced by diamide, Oxs1 or Pap1 plays a negative role with full de-repression requiring loss of both proteins. In a third class of genes, Oxs1 positively regulates expression, but in its absence, Pap1 plays a negative role. The Oxs1–Pap1 regulatory interaction appears evolutionarily conserved, as heterologous (human, mouse and *Arabidopsis*) Oxs1 and Pap1-homologues can bind interchangeably with each other *in vitro*, and at least in the fission yeast, heterologous Oxs1 and Pap1-homologues can substitute for *S. pombe* Oxs1 and Pap1 to enhance stress tolerance.

## INTRODUCTION

In oxidative stress, different oxidants can elicit different responses and the damages caused can be distinct (1). Whereas H_2_O_2_ oxidizes a thiol to sulfenic (SOH), then to sulfinic (SO_2_H) or sulfonic (SO_3_H) acid, as well as promoting disulfide formation between a SOH and a thiol (2), the reactive electrophilic species diamide specifically triggers disulfides between thiols (3), producing a subcategory of oxidative stress that is also referred to as disulfide stress (4).

**Figure 1.**
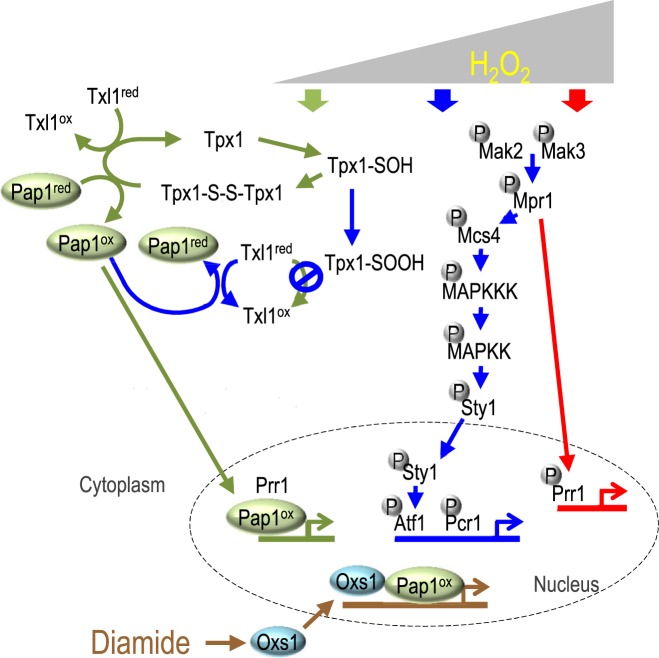
Model of transcriptional response to H_2_O_2_ or diamide (and Cd) and the stress induced nuclear localization of Oxs1. Low, medium and high concentrations of H_2_O_2_ induce pathways shown by green, blue and red arrows, respectively. Diamide or Cd induces the Pap1–Oxs1 pathway (brown arrows). Oxidized or reduced state indicated by superscript ox or red. Thiol, sulfenic, sulfinic and disulfide shown as SH, SOH, SOOH and S-S, respectively. Circled P: phosphorylated residue.

In brief, three signaling pathways respond to H_2_O_2_ in the fission yeast *Schizosaccharomyces pombe* (5,6) (Figure 1). At low H_2_O_2_ concentration, thioredoxin peroxidase Tpx1 is oxidized to its disulfide form, which leads to oxidization of the proteasome-associated thioredoxin-like protein Txl1, as well as Pap1 (7,8). Oxidized Pap1 relocates from the cytoplasm to the nucleus as it bypasses nuclear export by Crm1 (Exportin1), forms a heterodimer with transcription factor Prr1, and turns on the expression of target genes (9,10). At a higher H_2_O_2_ level, hyper-oxidized Tpx1 fails to oxidize Txl1, permitting reduced Txl1 to reduce Pap1 and turn off the early stress response (7,11). The Sty1–Atf1 pathway takes over with a phosphorelay system comprising of histidine kinase sensors Mak2 and Mak3 that passes the signal to the histidine-containing phosphorelay protein Mpr1, then to response regulator Mcs4, and finally through the MAPK cascade to the MAPK Sty1 (12). Sty1 then phosphorylates the basic region/leucine zipper motif (bZIP) transcription factor Atf1, which dimerizes with another bZIP protein Pcr1 to activate downstream targets (13,14). At high levels of H_2_O_2_, the phosphorylated Mpr1 is thought to activate Prr1 directly, which then activates genes responding to acute oxidative stress (15).

In contrast to the wealth of knowledge on H_2_O_2_ stress, less is known of the response to diamide. Previous studies have identified Pap1, as well as its budding yeast homologue Yap1, responding to diamide or various cytotoxic drugs and heavy metals by translocating from the cytoplasm to the nucleus to activate a stress response (9,16). In this paper, we describe Oxs1, a new player that interacts with Pap1 at the target promoters in a diamide or Cd-dependent manner. Moreover, heterologous Oxs1-like proteins can also enhance diamide stress tolerance in the fission yeast. *In vitro, S. pombe*, human and *Arabidopsis* Oxs1 can interchangeably bind Pap1 or Pap1 homologues from human (cJun) and *Arabidopsis* (bZIP10), suggesting that Oxs1 may be a component of an evolutionarily-conserved stress response pathway.

## MATERIALS AND METHODS

### Genetic materials

*S. pombe* strains include JS23 (WT, *h^+^ ura4.294 leu1.32*); TP108-3C (*h^−^ leu1 his22 ura4 pap1::ura4^+^*) (17); NT224 (*h^–^ leu1 ura4 sty1-1*) (18); JM1066 (*h^+^ leu1 atf1::ura4^+^*) (14); JX26 (*H^90^ ade6 leu1 ura4 pcr1::ura4^+^*) (14); and the JS23-derived strains created through homologous gene disruption (Supplementary Figure S1): *oxs1Δ; pap1Δ; pap1Δoxs1Δ; FLAG-oxs1; HA-pap1; FLAG-oxs1 HA-pap1; HA-pap1 oxs1Δ;* and *FLAG-oxs1 pap1Δ*. The cDNAs were inserted into pART1 or pSLF173, expressed from the *adh1* or *nmt1* promoter, respectively; further details in Supplementary Materials and Methods. Genetic manipulations performed according to the Fission Yeast Handbook (http://www.biotwiki.org/foswiki/bin/view/Pombe/NurseLabManual), using YES (rich) or EMM (minimum) growth media.

### RNA-Seq and qRT-PCR

Cells grown to OD_600_ 0.3 were treated with 1.0 mM diamide for 0, 20, 60, 90 and 120 min prior to harvest of total RNA (RNeasy Mini Kit, Cat# 74104, Qiagen). RNA-seq library construction and sequencing were carried out by BGI-Tech (Shenzhen, China); expression level calculated as RPKM was compared between diamide treated samples and untreated samples. For qRT-PCR, cells grown to OD_600_ 0.3 were treated with 1.5 mM diamide for 0–5 h before harvesting total RNA. Reverse transcription was conducted using PrimeScript™ RT reagent Kit (Cat# RR047A, TaKaRa); qRT-PCR with SYBR Premix Ex Taq™ Mix (Cat# DRR820A, TaKaRa) on LightCycler^®^480 II (Roche). Relative expression level normalized to *act1*^+^ (SPBC32H8.12c) of WT cells at 0 h.

### *In vitro* GST-pull down assay

Coding regions of *oxs1*^+^ or *pap1*^+^ were inserted in-frame into pGEX-4T-1 (Cat# 28954549 GE Healthcare Lifesciences) to generate GST fusion proteins or into pET-21d (Cat# 69743-3, Novagen) to generate His fusion proteins. *In vitro* GST pull-down assays were as follows: Cell lysates from *Escherichia coli* BL_21_ (DE_3_) pLysS (Cat# BL21C-24, GE) expressing GST fusion proteins were incubated with MagneGST™ beads (Cat# V8611, Promega) at 4°C for 4 h, followed by four washes with buffer (25 mM Tris–HCl pH 7.2, 150 mM NaCl, 1 mM EDTA, 1 mM EGTA, 1% Triton X-100, 0.5% NonidetP-40, 1 mM DTT). Beads bound with GST fusion proteins were incubated overnight at 4°C with cell lysates from *E. coli* BL_21_ expressing His fusion proteins, followed by four washes with buffer. Immunoprecipitates were subjected to western blotting (see Supplementary Materials and Methods).

### Co-immunoprecipitation analysis

Cells grown to OD_600_ 0.5 were collected and lysed in lysis buffer (50 mM Tris–HCl pH 8.0, 150 mM NaCl, 0.1% NonidetP-40, 0.1% SDS, 12 mM sodium deoxycholate) with glass beads in a bead beater (Fastprep-24 MP Biomedical). Lysate was centrifuged at 12,000 rpm for 15 min at 4°C. Protein extract was incubated with 5 μg polyclonal anti-HA antibody (Cat# ab9110, Abcam) overnight at 4°C, then a 25 μl mixture (1:1) of Dynabeads^®^ Protein A:Protein G (Cat# 10002D, Life Technologies AS) was added and incubated at 4°C for 4 h. After washing the beads 6x with buffer (50 mM Tris–HCl, pH 8.0, 150 mM NaCl, 1 mM EDTA, 0.1% NonidetP-40, 5% glycerol pH 7.4), the immunoprecipitates were eluted by boiling in SDS-PAGE loading buffer for western blotting using Monoclonal anti-HA antibody (Cat# ab16918, Abcam) and anti-FLAG antibody (Cat# F1804, Sigma).

### Chromatin immunoprecipitation

Cells were cross-linked by 1% formaldehyde for 20 min at 30°C, stopped with 125 mM glycine for 5 min, pelleted, washed twice with PBS (137 mM sodium chloride, 2.7 mM potassium chloride, and 11.9 mM phosphate buffer, pH 7.4), frozen immediately in liquid nitrogen and stored at -80°C. Nucleic acid extraction by enzymatic lysis was conducted according to the Fission Yeast Handbook. Chromatin were released and sheared to an average size of 500 bp using M220 sonicator (Covaris). The immunoprecipitation and DNA recovery procedures were as described (19) (Supplementary Materials and Methods). The immunoprecipitated DNA fragments were quantified by qPCR. Intergenic region of *S. pombe* chromosome I (position 465226–465326) used as negative control. Fold enrichment normalized against intergenic region of WT(HA-Pap1 knock-in) or WT strain under non-stress condition set as 1. All experiments were repeated three times.

## RESULTS

### A nucleocytoplasmic HMG protein mediates stress tolerance

**Figure 2.**
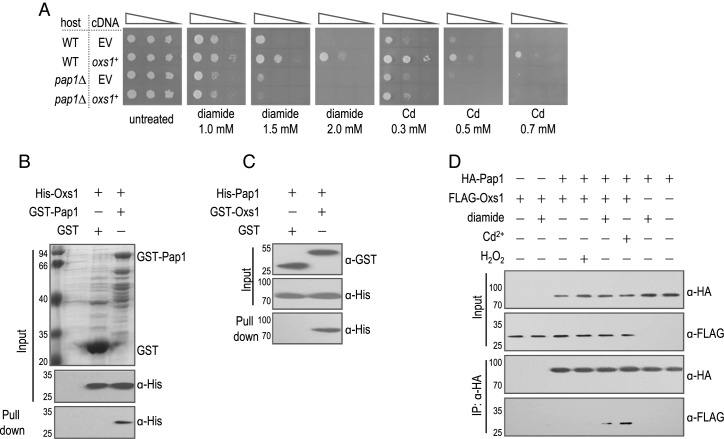
Oxs1-mediated diamide tolerance requires Pap1. (**A**) Cells of 10-fold serially diluted containing empty vector pART1 (EV) or pART1 expressing *oxs1*^+^ spotted on EMM selective media without or with diamide or Cd. (**B**) GST-Pap1 or GST bound to GST affinity resin incubated with His-Oxs1. Pull-down fractions analyzed by western blotting with anti-His antibody. (**C**) GST-Oxs1 or GST bound to GST affinity resin incubated with His-Pap1. Pull-down fractions detected with anti-His antibody. (**D**) Protein extracts from strain producing HA-Pap1, FLAG-Oxs1 or both were treated without or with diamide (1 mM, 3 h), Cd (0.1 mM, 3 h) or H_2_O_2_ (0.2 mM, 5 min), immunoprecipitated with anti-HA antibody and detected by anti-HA or anti-FLAG antibody. Input fractions represent 2% of His-tagged proteins in pull-down assays. Numbers left of panels B, C and D indicate size markers in kD.

Previously, we described using an *Arabidopsis* cDNA expression library to select clones that could enhance stress tolerance in *S. pombe* (20). Although we recovered an *Arabidopsis* cDNA corresponding to At1G16210, we decided to conduct the functional analysis with its *S. pombe* homolog SPBC29A10.12, which is predicted to encode a 207aa high mobility group (HMG) protein (Supplementary Figure S2). Expression of SPBC29A10.12 cDNA enhanced tolerance to diamide and Cd (Figure 2A), but not to H_2_O_2_, salt (NaCl or KCl), osmotic (sorbitol) or heat (42°C) stress (Supplementary Figure S3A). A deletion in SPBC29A10.12 created by homologous recombination (Supplementary Figure S1A) was indeed more sensitive to Cd and diamide but not to the other stresses (Supplementary Figure S1B). Since both diamide and Cd deplete the GSH pool; diamide promotes formation of GSSG and Cd sequesters GSH-derived (γ-Glu-Cys)_*n*_-Gly peptides in the vacuole (21), this gene may be specific for the subcategory of oxidative stress known as GSH or disulfide stress (4). Indeed, a lower GSH/GSSG ratio was found after treatment with diamide or Cd (Supplementary Figure S4) in WT and in the SPBC29A10.12*Δ* strain, but not when expressing the SPBC29A10.12 cDNA. Given its role in oxidative stress, we named SPBC29A10.12 *oxs1*^+^ for *oxidative stress 1*.

### Oxs1 requires and interacts with Pap1 in a stress-dependent manner

Of the three H_2_O_2_ stress pathways in *S. pombe*, the Mpr1–Prr1 pathway does not appear to play a role in diamide tolerance, as *prr1Δ* is insensitive to diamide (22). Since *sty1Δ* or *pap1Δ* is diamide sensitive (9), we investigated whether components of these pathways could be involved. Enhanced tolerance to diamide or Cd by the *oxs1^+^* cDNA was still observed in *sty1Δ, atf1Δ* or *pcr1Δ* strains (Supplementary Figure S3B), indicating that the Sty1–Atf1 pathway is not essential for Oxs1 function. However, enhanced tolerance to diamide or Cd was not observed in the *pap1Δ* mutant TP108-3C (Supplementary Figure S3B). To confirm, we generated our own *pap1Δ* strain (Supplementary Figure S1C) and the results were the same (Figure 2A). While this indicates that Oxs1 function requires Pap1, the converse does not hold, as overexpression of *pap1^+^* can rescue stress sensitivity in the *oxs1Δ* background (Supplementary Figure S3C). This suggests that Pap1 can function without Oxs1, at least when overproduced. An additive effect was also not found, as over-expression of both *pap1*^+^ and *oxs1^+^* failed to increase stress tolerance beyond what was achieved through *pap1^+^* overexpression alone (Supplementary Figure S3D), while the *oxs1Δ pap1Δ* double mutant is as sensitive to diamide or Cd as the *pap1Δ* mutant (Supplementary Figure S3E). This suggests that Pap1 plays a more dominant role than Oxs1 for diamide or Cd tolerance.

To test whether Oxs1 may interact with Pap1, we first incubated His-tagged Oxs1 with GST-tagged Pap1 or the GST-only control. The resin that bound GST-Pap1 pulled down His-Oxs1 (Figure 2B). However, since the yield of GST-Pap1 from *E. coli* was relatively low, we switched to using GST-tagged Oxs1 to test against His-tagged Pap1. Likewise, the data show that the two proteins interact, as the affinity resin that bound GST-Oxs1 also pulled down His-Pap1 (Figure 2C). To test if this binding occurs *in vivo*, the native *oxs1*^+^ and *pap1*^+^ genes were replaced with versions encoding FLAG-tagged Oxs1 and HA-tagged Pap1, respectively (Supplementary Figure S1D and E). The tolerance phenotype of each single or double knock-in strain was indistinguishable from WT (Supplementary Figure S1F). Crude extracts immunoprecipitated with anti-HA antibody detected anti-FLAG cross reaction when cells were treated with diamide or Cd (Figure 2D), but not when cells were untreated or treat under conditions typically described for H_2_O_2_-induced Pap1 nuclear localization (10). This shows that the Oxs1–Pap1 interaction is specific for diamide or Cd.

### Oxs1 is a co-regulator of Pap1

**Figure 3.**
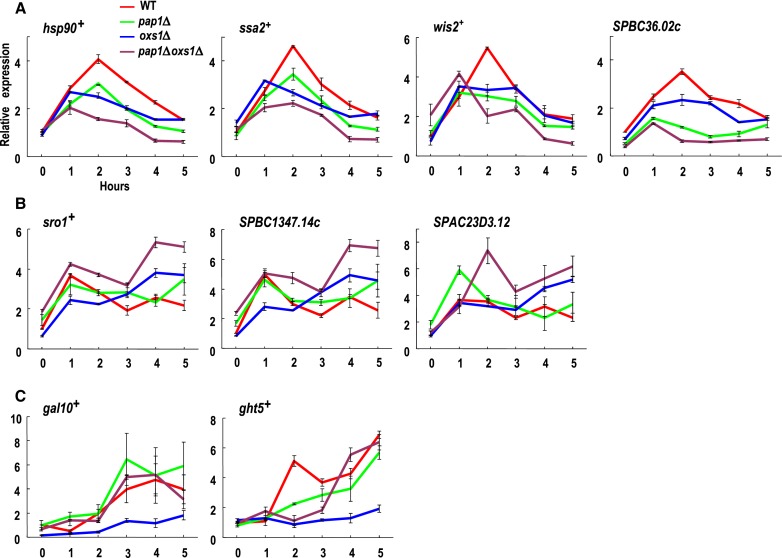
Transcription profiles of diamide-induced genes in WT and mutants. (**A–C**) qRT-PCR of mRNA from cells grown in EMM media treated with 1.5 mM diamide for 0–5 h; beta-actin (*act1*) mRNA used for normalization. Relative expression for each gene in each genotype normalized to WT at 0 h set as 1. Error bars show SEM from three independent experiments. Data from non-synchronized dividing cells.

Since Pap1 is a transcription factor, and it interacts with Oxs1 *in vivo*, we considered the possibility that they could interact at target gene promoters. To find potential target genes for these proteins, we conducted an RNA-seq analysis. This led to finding 307 genes induced by 2-fold or more in cells grown in diamide (Cd not tested). Of these, gene annotation analysis shows that 101 of them are related to the stress response, protein folding, transport or transcription. Among the 101, 28 were induced by more than 4 fold. However, only 20 of the 28 genes showed reproducible induction by diamide in a second test by qRT-PCR (Supplementary Table S1). Hence, these 20 genes were selected for qRT-PCR in the WT, *pap1Δ, oxs1Δ* or *pap1Δoxs1Δ* strains. Their expression patterns during the 5 hours of diamide treatment place them into four classes. Class I with 4 members (*hsp90*^+^, *ssa2*^+^, *wis2*^+^, *SPBC36.02c*) showed reduced transcription in either a *pap1Δ* or *oxs1Δ* background and lowest expression in the *pap1Δoxs1Δ* double mutant (Figure 3A). This indicates that while each protein alone enhances transcription, the presence of both Pap1 and Oxs1 exerts an additive positive effect. Class II with 3 members (*sro1*^+^, *SPBC1347.14c, SPAC23D3.12*) were unaffected by the *pap1Δ* or *oxs1Δ* mutation, but showed elevated expression in the *pap1Δoxs1Δ* background (Figure 3B), suggesting that either Oxs1 or Pap1 alone is sufficient to repress transcription, and that de-repression requires loss of both proteins. Class III with two members (*gal10*^+^, *ght5*^+^) showed lower expression in the *oxs1Δ* strain (Figure 3C), which suggests that Oxs1 positively regulates expression. However, an expression pattern similar to that of the WT was found in the *pap1Δoxs1Δ* or *pap1Δ* genotype. This pattern may indicate that in the absence of Oxs1, the lower expression may be due to Pap1 playing a role in negative regulation. Relieve of this Pap1-mediated repression is seen in the presence of Oxs1, as in the WT, or in the absence of Pap1, as in the *pap1Δ* or *pap1Δoxs1Δ* genotype. Class IV with 11 members (Supplementary Table S1) showed expression patterns that appear unaffected by the *pap1Δ, oxs1Δ*, or *pap1Δoxs1Δ* genotype, and hence their diamide induction must be due to stress pathways unrelated to Pap1 or Oxs1.

### Effective Pap1 binding to target promoters requires diamide and Oxs1

**Figure 4.**
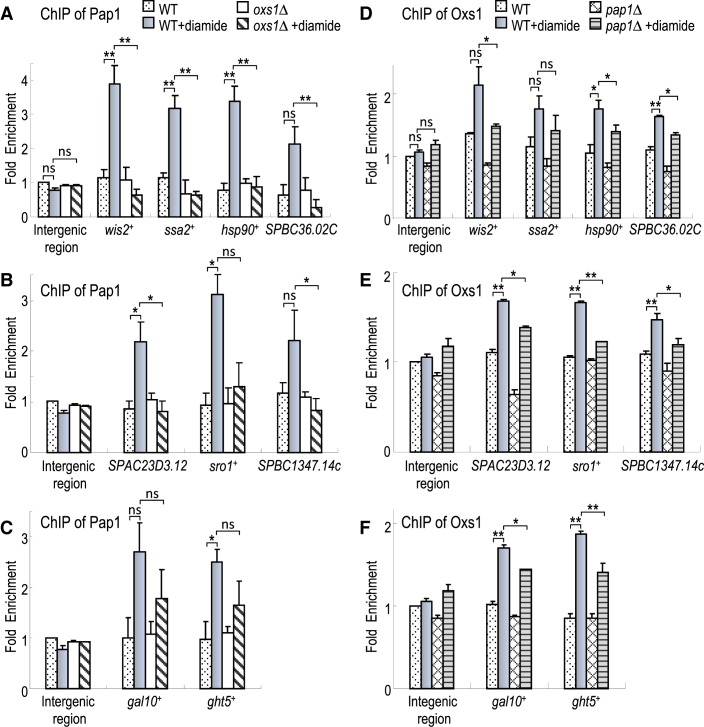
Oxs1 and Pap1 cooperative binding to target promoters. (**A–C**) ChIP-qPCR of Pap1 binding to gene promoters in WT(HA-Pap1 knock-in) or *oxs1Δ*(HA-Pap1 knock-in) strains producing HA-Pap1 treated 5 h without or with 1.5 mM diamide. HA antibody used to immunoprecipitate HA-Pap1. (**D–F**) ChIP-qPCR of Oxs1 binding to gene promoters in WT or *pap1Δ* strain treated as in (A–C). Anti-Oxs1 polyclonal antiserum used to immunoprecipitate Oxs1; antiserum specificity shown in Supplementary Figure S5. Intergenic region of *S. pombe* chromosome I (position 465226 to 465326) used as negative control. Fold enrichment normalized against intergenic region of WT(HA-Pap1 knock-in) strain (A–C) or WT strain (D–F) under non-stress condition set as 1. SEM (error bars) from three independent experiments. *P < 0.05 and **P < 0.01 using unpaired Student's *t* tests, ns: no significant effect at *P* < 0.05 level.

As a transcription factor, it is likely that Pap1 binds these diamide-induced promoters. To test this possibility, ChIP was conducted on the HA-Pap1 strain with anti-HA antibody followed by qPCR of the gene promoters. The data show that for all nine genes, above background amplification of their promoters was detected only when WT cells were exposed to diamide (Figure 4A–C), indicating that Pap1 is recruited to these promoters during diamide stress. When the ChIP-qPCR analysis was conducted in the *oxs1Δ* strain, diamide-induced Pap1 binding was abolished for the Classes I and II genes, suggesting that Oxs1 is necessary for Pap1 binding to these promoters (Figure 4A and B). For the Class III genes, Pap1 promoter binding in response to diamide was found in the *oxs1Δ* genotype, but at lower efficiency than in a WT background (Figure 4C). This above background level of Pap1 binding is consistent with an interpretation that in the absence of Oxs1, Pap1 exerts a repressive effect on these genes, and this repression is presumed to require promoter binding.

### Oxs1 binds to the same promoters induced by diamide

Since Oxs1 is necessary for Pap1 binding to target promoters, we tested if Oxs1 would also bind to the same promoters. ChIP was performed using anti-Oxs1 antibody followed by qRT-PCR. Above background amplification of these nine promoters was detected only when cells were exposed to diamide (Figure 4D–F). This effect was not significant in an *oxs1Δ* strain, showing that the anti-Oxs1 antibody has specificity for Oxs1 (Supplementary Figure S5). In a *pap1Δ* strain, Oxs1 still responds to diamide with more effective binding to nearly all of these promoters, but less effective than in the WT (*pap1*^+^) background. This suggests that as with Pap1, Oxs1 is recruited to each of these promoters during diamide stress, and that the presence of Pap1 makes Oxs1 binding to these targets more effective.

### Oxs1 and Pap1 relocate to the nucleus during diamide or Cd stress

**Figure 5.**
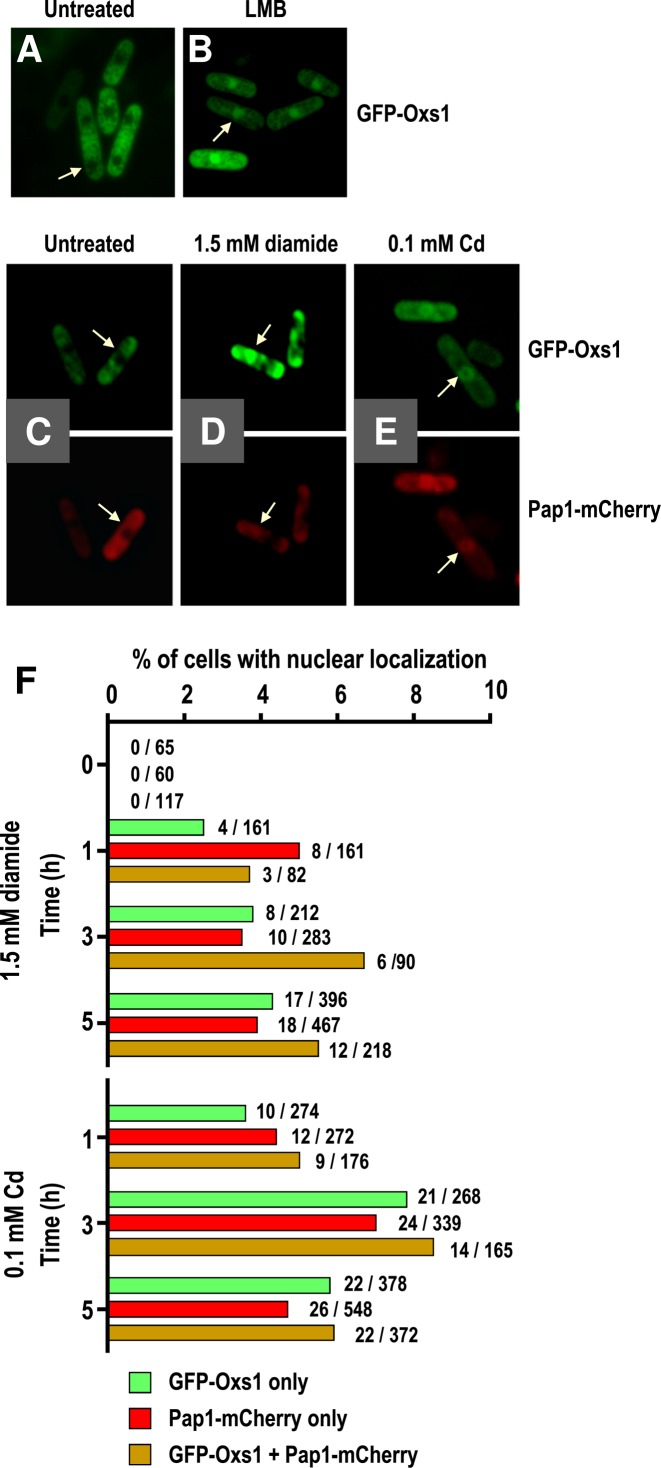
Stress-induced nuclear localization by Oxs1 and Pap1. Representative fluorescence microscopy of GFP-tagged Oxs1 or mCherry-tagged Pap1 fusion protein. Plasmid pART1-produced GFP-Oxs1 in WT cells treated without (**A**) or with (**B**) LMB. (**C–E**) Plasmid pART1-produced GFP-Oxs1 or pSLF173-produced Pap1-mCherry without or with treatment of 1.5 mM diamide or 0.1 mM Cd for 1 h. (**F**) Percentage of cells scored as GFP only, as mCheery only, or as having both GFP and mCherry signals in the nucleus. Number of cells scored positive over total cells counted shown to the right of each bar.

The protein subcellular localization program PSORT: http://psort.ims.u-tokyo.ac.jp/ predicts a tripartite NLS consisting of aa residues 4–10 (PKKRAEK), 23–26 (KKKK) and 129–135 (PERRFKA) as well as a nuclear export signal (NES) between aa107 to aa116 (IDDALDLLSL, conserved aa underlined; Supplementary Figure S2). This suggests that Oxs1 might be a nucleocytoplasmic shuttling protein regulated by the exportin Crm1. When Oxs1 was fused to the C-terminus of the green fluorescence protein GFP and inserted behind the *adh1* promoter on a multi-copy plasmid, the GFP signal was indeed excluded from the nucleus (Figure 5A). However, when the cells were treated with leptomycin B (LMB), a *Streptomyces* metabolite that blocks Crm1 interaction with the NES of a cargo protein (23), the GFP-Oxs1 protein could be found in the nucleus (Figure 5B).

As Figure 3 shows that target promoters are regulated by Oxs1 and Pap1, we examined their subcellular localization under the same treatment conditions. With plasmid-encoded GFP-Oxs1 and Pap1-mCherry fusion proteins, cells without exposure to stress showed an absence of fluorescence in the nucleus (Figure 5C). When treated with diamide or Cd, green and/or red fluorescence could be detected in the nucleus in a small percentage of the cell population (Figure 5D and E). Roughly the same percentage of cells scored positive for nuclear detection of GFP, mCherry or both (Figure 5F). This is consistent with the deduction that both proteins act in concert. However, since scoring positive for nuclear localization in this assay rests on having a sizable percentage of the signals in the nucleus, the low percentage of cells scoring as positive could be explained by the abnormally high amounts of proteins in these overexpression strains. The corollary would be that even in cells with a small percentage of total Oxs1 and Pap1 in the nucleus, and hence counted as predominantly cytoplasmic GFP-Oxs1 and Pap1-mCherry, there could be sufficient Oxs1 and Pap1 to cause the effect on target gene expression shown in Figure 3.

### Overexpression of target genes for stress tolerance

For the four Class I genes up-regulated by Pap1 and Oxs1, their role in diamide tolerance was examined by expressing each individually in a multi-copy plasmid. Every gene was able to enhance tolerance to diamide in the WT background (Supplementary Figure S3F), although not as effective as overexpressing their up-stream regulator *oxs1^+^* or *pap1^+^*. Two of them, *wis2*^+^ and *hsp90*^+^, also showed some enhanced tolerance in the *oxs1Δ* mutant, but none was effective in a *pap1Δ* background (Supplementary Figure S3G,H). Hence, the loss of Pap1, presumed to activate many other genes that respond to diamide, cannot be compensated through the simple overexpression of any one of these downstream targets.

### Oxs1 stress tolerance function conserved among eukaryotic species

BLAST analysis indicates that homologs of deduced Oxs1 are present in a wide range of eukaryotes including human, mouse, rat, zebrafish, fruitfly, mosquito, nematode, *Neurospora*, rice, maize, and *Arabidopsis*, but surprisingly not in the budding yeast *Saccharomyces cerevisiae* (Supplementary Figure S2). Previously described as a family of unknown proteins named DUF1014 (IPR010422), there has been a recent report of a human member of this family, Ccdc124 (Coiled-Coil Domain Containing Protein 124), identified as a novel component of the centrosome during interphase and G2/M transition and is involved in cytokinesis (24).

**Figure 6.**
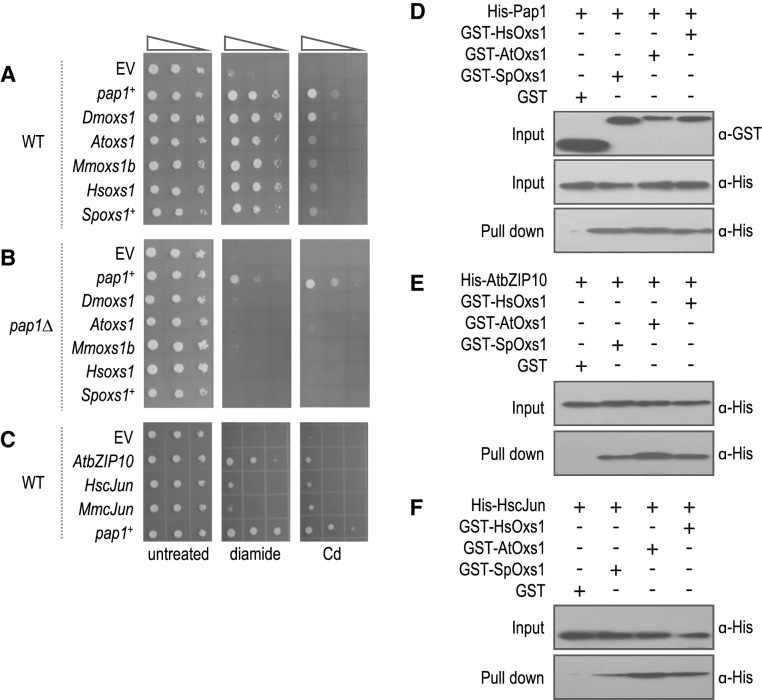
Heterologous Oxs1 or Pap1 enhance stress tolerance and interact *in vitro*. (**A–C**) 10-fold serially diluted cells containing pART1 (EV) or pART1 expressing indicated cDNAs on EMM media without or with 2 mM diamide or 0.7 mM Cd. Photos taken after 5 days of incubation. (**D–F**) Western blots using anti-GST or anti-His antibodies. GST-HsOxs1, GST-AtOxs1, GST-SpOxs1 or GST control incubated with His-Pap1 (D), His-AtbZIP10 (E) or His-HscJun (F). Input control shown for GST done at the same time for (D-F).

Conservation of regulatory components for oxidative stress has long been noted among diverse organisms. For instance, *S. pombe* Sty1 is similar to human and mouse Jun-NH_2_-terminal kinase (JNK) and p38 kinase (18). *S. pombe* Atf1 is most homologous with human and mouse ATF-2 (25) and *S. pombe* Pap1 with human and mouse cJun (26). In *Arabidopsis*, however, MAPKs AtMPK3 and AtMPK6 activate plant-specific WRKY transcription factors (27,28), but there is evidence for bZIP transcription factors involved in pathogen stress (29). To ask whether there is functional conservation among heterologous Oxs1 proteins, cDNAs encoding the Oxs1 homologues from *Arabidopsis* (AtOxs1, At1G16210), fruitfly (DmOxs1, Dmel_CG6013), mouse (MmOxs1b, NP_081240) and human (HsOxs1, hCG_2000823) were found to be as effective as SpOxs1 for tolerance against diamide or Cd (Figure 6A), but not to H_2_O_2_, salt, osmotic or heat (Supplementary Figure S3A). Moreover, these homologue proteins also failed to enhance diamide tolerance in the *pap1Δ* genotype, indicating that Oxs1 homologues also require *S. pombe* Pap1 for function (Figure 6B).

Since *S. pombe* Pap1 is not present in the other eukaryotes, heterologous Oxs1 proteins might use Pap1-like proteins as co-activators, and if so, those Pap1-like proteins might behave similarly in *S. pombe*. When AtbZIP10 (At4g02640), along with human and mouse cJun, were tested in *S. pombe*, AtbZIP10 was nearly as effectively as Pap1 for diamide tolerance, and a weaker effect against Cd. The human and mouse cJun homologs also showed weak but positive effects for diamide and Cd (Figure 6C).

Physical interaction among heterologous Oxs1 and Pap1-like proteins was also detected by the *in vitro* binding assay. GST-tagged Oxs1, HsOxs1, AtOxs1 or the control GST protein was incubated with His-tagged *S. pombe* Pap1, purified through GST affinity resin, and probed with anti-His antibody. Like SpOxs1, HsOxs1 and AtOxs1 were effective in binding *S. pombe* Pap1 (Figure 6D). Likewise, when AtbZIP10 or HscJun was used instead of Pap1, the GST-Oxs1 protein from human, *Arabidopsis* or *S. pombe* each showed interaction (Figure 6E and F).

## DISCUSSION

Of the many regulatory proteins identified in the oxidative stress response to H_2_O_2_, only loss of Sty1 and Pap1 affected diamide tolerance (9), and of the two, only Pap1 is necessary for Oxs1-mediated tolerance to diamide and Cd. Oxs1 and Pap1 can bind to each other, and moreover, this interaction is found only in cells grown with diamide or Cd, but not H_2_O_2_. We did not test further with Cd, but with diamide, the ChIP assays show that the two proteins bind the same selected set of gene promoters that are diamide induced. Moreover, this binding only occurs during diamide stress. Although it may be possible that Pap1 directs Oxs1 to the target promoters, the ChIP data suggest otherwise, that Oxs1 binds these same promoters even in the absence of Pap1. On the contrary, Pap1 binding to Class I and Class II promoters requires Oxs1, as though Oxs1 is directing the diamide stress response. The one conflicting data is that in the absence of Oxs1, Pap1 can still enhance diamide tolerance. It is possible that this effect is caused by an overproduction of Pap1 such that it either overrides the need for an Oxs1 partner, or that Pap1 also (hyper) activates other stress ameliorating genes that are not dependent on Oxs1.

Although Pap1 is well recognized to activate transcription, this role was found only with the Class I genes. With the Classes II and III genes, its role appears to be that of a negative regulator. The Classes I to III genes show that regulation by Pap1 and Oxs1 can be complex, requiring one or both proteins to be either positive or negative regulators. However, in all cases, the presence of one protein appears to help recruit the other to the same promoter. Oxs1 did not require Pap1 to bind to the target promoters, but was less effective in its absence. For the Class IV genes, in which we have not found convincing linkage to Pap1 and Oxs1, it remains possible that Sty1 may be involved in some of them, although the Sty1 downstream regulator is not likely to be Atf1 or Pcr1.

In summary, we have added a new Pap1–Oxs1 pathway in the oxidative stress response in *S. pombe* for diamide (Figure 1) that appears specific for disulfide or GSH stress, and this pathway also responds to Cd most likely because it also causes the GSH depletion effect of diamide (Supplementary Figure S4H). In the absence of stress, Crm1 (exportin1) keeps Oxs1 in the cytoplasm. During disulfide stress, Oxs1 moves to the nucleus. For some promoters, Oxs1 recruits Pap1 for co-activation (Class I) or co-repression (Class II). For other targets, Oxs1 neutralizes the repressive effect of an existing Pap1 (Class III). In this Pap1–Oxs1 pathway, Pap1 is presumed to be in an oxidized state in the nucleus, as has been experimentally shown with the budding yeast homologue Yap1 (30) or when *S. pombe* is treated with diethylmaleate (another glutathione-depleting agent) or H_2_O_2_ (31,32). Of particular interest is that since Oxs1-homologous proteins, as well as Pap1-homologous proteins are found in diverse eukaryotes from fungi, insects, worms, plants to mammals, it is likely that the Oxs1–Pap1 co-regulatory system is evolutionarily conserved. Consistent with this hypothesis, heterologous Oxs1 and Pap1-homologues can bind each other *in vitro*, and at least in the fission yeast, they can also pheno-copy *S. pombe* Oxs1 and Pap1 for stress tolerance.
